# Vector-borne disease surveillance and natural disasters.

**DOI:** 10.3201/eid0402.980227

**Published:** 1998

**Authors:** R S Nasci, C G Moore

**Affiliations:** Centers for Disease Control and Prevention, Fort Collins, Colorado 80522, USA.


### Vector-borne Disease Surveillance and Natural Disasters

Natural disasters, such as hurricanes and floods, are frequently followed by a proliferation of mosquitoes and requests from residents and government agencies for widespread application of insecticides. However, nuisance mosquito species, which often require emergency control measures, rarely present a threat to public health (1). Natural disasters in the continental United States have rarely been accompanied by epidemics of mosquito-transmitted disease (2). Nevertheless, public health disaster response policies should include a provision for monitoring increases in the prevalence of potentially infectious mosquitoes and the risk for arboviral disease in the affected areas.

Four major arboviruses (viruses transmitted by mosquitoes or other arthropods) are of human and veterinary public health importance in the United States: eastern equine encephalomyelitis (EEE), western equine encephalomyelitis (WEE), St. Louis encephalitis (SLE), and LaCrosse (LAC) encephalitis. Because of its unique ecology, LAC virus does not occur in an epidemic form and would not increase as a result of floods or hurricanes. The three viruses of primary concern (EEE, SLE, WEE) overlap in their distribution, but each has a distinct ecology, involving different mosquito species and avian amplifier hosts (3)[1]. Despite these differences, populations of primary or secondary vector species of each virus may increase significantly in response to heavy rainfall or flooding. Therefore, under certain circumstances, disasters might produce increases in disease risk.

Several major floods and hurricanes have been evaluated since 1975 (Table). The major geographic and ecologic regions of the United States, some of which contain each of the common epidemic arboviruses (EEE, SLE, WEE), are included. With the exception of the Red River flood of 1975, disasters have not increased transmission of arboviruses to humans or domestic animals. The single case of EEE associated with Hurricane Fran in 1996 may have been acquired before the hurricane. Similarly, cases of EEE and WEE in domestic animals, especially horses and emus, do not appear to increase after floods or hurricanes (SLE does not cause disease in most domestic animals).

Despite the fact that epidemics of arboviral encephalitis have rarely followed hurricane- or flood-related disasters in the United States, these events can increase the risk for human arboviral disease in certain circumstances. In nine of the ten events in which surveillance has been conducted (Table), arbovirus activity was detected in surveillance programs initiated after the event, which indicates the existence of an enzootic transmission cycle in the area. Under these circumstances, increases in vector mosquito population density can enhance transmission and increase the prevalence of potentially infectious mosquitoes, which in turn increases the risk for human disease, particularly when coupled with expanded human exposure to mosquitoes after a disaster (e.g., residents and recovery workers removing debris, restoring housing, and living in substandard conditions).

Public health policies dealing with flood-related disasters must include provisions addressing mosquito-transmitted disease. Preferably, data from an existing arbovirus surveillance program can be evaluated to determine the status of transmission cycles before the event and determine the risk for disease. For example, California's long-term surveillance has established a baseline to which post-disaster virus activity can be compared (4-7). After the 1994-95 winter flood in California, Moore (unpub. data) used those data to determine that WEE seroconversion rates in sentinel chickens did not differ from rates in previous years. However, in most hurricane- and flood-prone areas, routine surveillance is not conducted.

In the absence of an existing program, arbovirus surveillance should be initiated as part of the disaster response plan. At the very least, these programs should determine population indexes of key vector species, virus infection rates in those species, and seroprevalence in sentinel or wild animals. Additional useful information

[1]Additional information may be obtained at http://www.cdc.gov/ncidod/dvbid/arbor/arboinfo.htm

Table. Natural disasters in the continental United States since 1975[a]

| Year | State/region | Event | Surveillance done? | Activity detected? | Human cases | Veterinary cases |
|---|---|---|---|---|---|---|
| 1975 | N.D., Minn. | Red River flood | Yes | WEE[b] in mosquitoes | 55 WEE, 12 SLE[c] | 281 WEE (estimated) |
| 1989 | S.E. United States | Hurricane Hugo | Yes | EEE[d] in mosquitoes | None | (No data) |
| 1992 | Fla., La. | Hurricane Andrew | Yes | None | None | None |
| 1993 | Ariz. | Gila River flood | Yes | SLE, WEE in mosquitoes | None | None |
| 1993 | Midwestern United States | Mississippi, Missouri river flooding | Yes (7 states) | WEE - S.D., SLE - Il. | None | None |
| 1994 | Ala., Fla., Ga. | Tropical storm Alberto | Yes | EEE-Al, Fl | None | EEE in horses, emus - Al, Fl |
| 1995 | Calif. | Winter & spring floods | Yes | WEE, SLE in sentinel flocks | None | WEE |
| 1996 | Calif. | Winter flood | Yes | WEE, SLE in chickens, WEE in mosquitoes | None | None |
| 1996 | Oregon, Wash. | Winter flood | No | No | None | None |
| 1996 | N.C. | Hurricane Fran | Yes | EEE in mosquitoes | 1 EEE | EEE in horses |
| 1997 | Colo. | Summer floods | Yes | WEE in chickens | None | None |
| 1997 | N.D., Minn. | Red River flood | Sporadic | None reported | None | None |

[a]Surveillance data collected by the Division of Vector-Borne Infectious Diseases, Centers for Disease Control and Prevention (CDC). State and local health departments assisted during emergency response. Federal Emergency Management Agency and Emergency Response Coordination Group, National Centers for Environmental Health, CDC, provided field support.
[b] Western equine encephalitis
[c] St. Louis encephalitis
[d] Eastern equine encephalitis includes prevailing weather conditions, time of year, human exposure, human population at risk, historical patterns of arboviral disease, and the potential for imported diseases (3).

**Roger S. Nasci and Chester G. Moore**
Centers for Disease Control and Prevention, Fort Collins, Colorado 80522, USA

### References

1. Centers for Disease Control and Prevention. Emergency mosquito control associated with Hurricane Andrew-Florida and Louisiana, 1992. MMWR 1993;42:240-2.
2. Centers for Disease Control and Prevention. Rapid assessment of vectorborne diseases during the Midwest flood-United States, 1993. MMWR 1994;43:481-3.
3. Moore, C.G., R.G. McLean, C.J. Mitchell, Nasci RS, Tsai TF, Calisher, CH, et al. Guidelines for arbovirus surveillance programs in the United States. Fort Collins, CO: U.S. Dept. of Health and Human Services, Centers for Disease Control and Prevention; 1993.
4. Emmons RW, Ascher MS, Dondero DV, Enge B, Milby MM, Hui LT, et al. Surveillance for arthropod-borne viral activity and disease in California during 1991. In: Proceedings of the California Mosquito and Vector Control Association; 1992.
5. Emmons RW, Ascher MS, Dondero DV, Enge B, Reisen WK, Milby MM, et al. Surveillance for arthropod-borne viral activity and disease in California during 1992. Proceedings of the California Mosquito and Vector Control Association; 1993.
6. Emmons RW, Ascher MS, Dondero DV, Enge B, Reisen WK, Milby MM, et al. Surveillance for arthropod-borne viral activity and disease in California during 1993. Proceedings of the California Mosquito and Vector Control Association; 1994.
7. Reeves WC. Epidemiology and control of mosquito-borne arboviruses in California, 1943-1987. Sacramento, CA: California Mosquito and Vector Control Association; 1990.
